# Professionalism, professional identity and community pharmacy culture: The context of substance dependency through the lens of student and early career pharmacists

**DOI:** 10.1111/add.70180

**Published:** 2025-09-25

**Authors:** Natalie Weir, Emma Dunlop, Adrian MacKenzie, Thomas Byrne, Katie Johnston, Alice O'Hagan, Zohaib Rehman, Holly Richardson, Aalia Shah, Gemma Wilson, Andrew Radley

**Affiliations:** ^1^ Strathclyde Institute of Pharmacy and Biomedical Sciences University of Strathclyde Glasgow UK; ^2^ Healthcare Improvement Scotland Glasgow Scotland UK; ^3^ School of Health Sciences University of Dundee Dundee UK

**Keywords:** community pharmacy, opioid agonist therapy, professional identity, professionalism, stigma, substance use

## Abstract

**Aims:**

This study aimed to explore the reflections of student and newly qualified pharmacists (NQPs) surrounding community pharmacy culture around substance dependency. This study explored professionalism and professional identity formation, and the possibility that a fragmented professional identity may impact behaviours and the provision of compassionate care.

**Design:**

Qualitative study: semi‐structured interviews were conducted with student and exploring stigma within community pharmacy environments in relation to people with substance dependency, the community pharmacy culture and their own ideas of professionalism and their professional identity formation. Interviews were undertaken by six pharmacy student researchers, under the supervision of two experienced researchers.

**Setting:**

Community pharmacies across Scotland.

**Participants:**

Twenty‐eight participants were recruited, including undergraduates based at Scottish Schools of Pharmacy (*n* = 20); Foundation Year Pharmacy students (*n* = 2) and NQPs (*n* = 6). Recruitment utilised university networks and social media platforms.

**Measurements:**

Interviews were conducted between September and November 2023 on Microsoft Teams®, each lasting 17–60 minutes. Data underwent inductive thematic analysis via NVivo® through data familiarisation, initial coding, theme searching, reviewing and defining and reporting.

**Findings:**

Stigmatisation of people with substance dependency attending a pharmacy was a prominent observation. This included negative stereotyping, adverse treatment because of judgements made about substance use and structural stigma relating to barriers to accessing care. Positive care provision in pharmacies was evident. Pharmacy staff who were empathetic, respectful, professional and who formed long‐term relationships with people with substance dependency were valuable role models for students and influenced their professional identify formation. Students appreciated the exposure to practice and the opportunity to make judgements that would mould the type of pharmacist they aspired to become. A number of participants reported that their university course poorly prepared them for the reality of supporting people with substance dependency.

**Conclusions:**

Pharmacy practice in Scotland appears to be characterised by stigma and lack of professionalism towards people with substance dependency, although there are examples of compassionate care. Observing staff in practice allowed participants of this study to develop their own professional identity and attitudes, yet there is a need to better prepare students in undergraduate curricula.

## INTRODUCTION

Around 31 million people used opiates globally in 2020, and the World Health Organization (WHO) estimated that in 2019 there were 600 000 deaths attributable to drug use, with 80% related to opioids [1]. In 2021 it was estimated that globally 463 360 people died from illicit drug use, 128 558 of whom died through illicit opioid use [2]. In Scotland there were 1172 deaths resulting from drug misuse in 2023, an increase of 12% from 2022 [3]. In the same year it was estimated that opioid agonist therapy (OAT) was prescribed to 30 190 people in Scotland [4]. The provision of OAT is widely accepted by patients and the general public as an integral part of community pharmacy services [5], with community pharmacies viewed as important and accessible primary care providers [6]. A recent survey in Glasgow, Scotland, identified that all pharmacies (*n* = 379) offered a supervised consumption service for methadone and alternative OAT choices [7]. Over half of pharmacies provided OAT to more than 30 patients, including 21% who supplied OAT for more than 60 patients. Successful OAT provision is associated with reduced mortality and morbidity for recipients and offers a route to recovery from drug dependence [8]. Additional harm reduction services are also delivered from community pharmacies, with approximately one‐quarter of pharmacies providing injecting equipment and related paraphernalia to reduce infections and the spread of blood‐borne viruses [5]. However, internationally, it is known that pharmacists hold varying beliefs towards supporting harm reduction associated with drug use [9, 10], with some pharmacists remaining disengaged, which can negatively affect equitable access to naloxone and needle provision [9, 11, 12, 13, 14].

Health services across the world have documented that stigma can readily be identified within their systems [15], which may impact the quality, extent and enthusiasm of care provided [9, 16]. Stigma has been described as ‘labelling, stereotyping, separation, status loss and discrimination in a context in which power is exercised’ [17]. Stigma has a profound effect on people with substance dependency and may jeopardise their chances of recovery [17]. One review identified substance dependency as one of the top three global health conditions that have various interventions developed to reduce associated stigma [15]. One of the most valued attributes of a community pharmacy service, from the perspective of people who inject drugs, has been described as the opportunity to be treated with dignity and respect [18]. Despite this, stigma associated with community pharmacy services has been documented and may arise from: the attitudes of staff; the interactions with other pharmacy customers; self‐stigmatisation; and the procedures and policies implemented for delivering the pharmacy services within a retail and business environment [7]. It has also been argued that stigma itself can be a main driver for health inequalities and mortality at a population level [19].

Stigmatising behaviours, attitudes and practices are the antithesis of professional practice and the standards underpinning the role of pharmacy professionals. When a patient walks into a community pharmacy, they expect to be met with expertise, care and respect by the professionals present [20]. Professional identity is a social construct that enables individuals to understand who they are in the context of their chosen profession, and it is a critical aspect of patient‐centred and compassionate care [21]. Professional identity formation involves internalising and demonstrating the behavioural norms, standards and values of a professional community, such that one comes to ‘think, act and feel’ like a member of that community. Professional identity influences how a professional perceives, explains, presents and conducts themselves [22]. Although further research is needed on the professional identity formation of pharmacy students [23], pharmacy students may start to form their professional identity on the day they begin their undergraduate studies [24], and within other fields there is evidence that professional identity begins to form before entry into formal higher education [23].

In terms of the professional identity of practicing pharmacists, a recent review illustrated that some pharmacists do not uniformly perceive themselves to be clinicians [25]. The dissonance caused by the uncertainty in professional identity may form some of the explanation for the self‐perception of untapped potential, underutilised skills and systematic underappreciation of pharmacists [26]. This may influence the professional and clinical support offered toward vulnerable populations, such as people with substance dependency. Pharmacy students, through experiential learning and part‐time work in community pharmacies [23], are likely to engage with people with substance dependency as this is the clinical setting of OAT across many nations. A fragmented and confused professional identity, which is considered common amongst pharmacists [27], may perpetuate and facilitate the provision of care that is stigmatising and discriminatory towards people with substance dependency.

The standards for the initial education and training of pharmacists in Scotland are defined by the regulator, the General Pharmaceutical Council (GPhC) [28]. These professional standards are included in undergraduate pharmacy programmes to support aspiring pharmacists to implement them in practice. Student pharmacists rely on experiential learning, part‐time employment and summer placements to observe pharmacists practicing their profession [29]. These opportunities provide scope for learned behaviours to arise. Personal encounters allow student pharmacists to witness how pharmacy professionals act, which influences how they endeavour to behave when they encounter similar situations in the future [29]. This experience provides students with opportunities to assess and evaluate professional values, attitudes and culture within a range of practice environments; thus, allowing the formation of their professional identity as a future pharmacist. The aim of this study was to explore the observations and reflections of student and newly qualified pharmacists (NQPs) in Scotland in relation to professionalism and professional identity formation within the context of the community pharmacy culture around substance dependency. This study explores the possibility that a fragmented professional identity may impact behaviours in community pharmacy practice affecting the quality and enthusiasm of the care provided, which could impact the provision of compassionate care for people with substance dependency. The authors aim to explore how pharmacy students and NQPs perceive and reflect on these dynamics to inform future education and professional development.

## METHODS

This qualitative study adheres to the Consolidated Criteria for Reporting Qualitative Research (COREQ) checklist (Appendix S1). This research was undertaken by six pharmacy student researchers, under the supervision of experienced researchers.

### Research material development

A participant information sheet (PIS), explaining the purpose of the research, as well as a consent form and demographic questionnaire were hosted on Qualtrics^XM^ (Qualtrics, Seattle, WA/Provo, UT, USA). A semi‐structured interview schedule was devised by the lead researchers (N.W. and E.D.), reviewed by A.R. and A.M., and underwent final review with six pharmacy student researchers. The interview schedule was devised with reference to Scotland’s Medication Assisted Treatment (MAT) standards for OAT and trauma‐informed care principles [30, 31], and focused on stigma, pharmacy culture, professionalism and identity formation. The interview schedule was piloted by E.D. with an NQP, and the recording was then viewed by the pharmacy student researchers as training and to agree the final schedule (Appendix S2).

### Sampling and recruitment

Participants were eligible if they were: pharmacy students from the schools of pharmacy in Scotland [either University of Strathclyde (UoS) or Robert Gordon University (RGU)]; Foundation Year Pharmacists (FYPs) on a final experiential learning year before qualifying; or NQPs who qualified in the last 3 years and were working within community pharmacy. Students from all years of study within university were eligible for inclusion, as professional identity formation is likely to begin within the early stages of education [23, 24]. Those excluded were the pharmacy student researchers themselves and students/pharmacists training or practicing outside of Scotland. Purposive sampling was undertaken to ensure balanced representation from both schools of pharmacy and variation in demographic characteristics, with an intended sample of five participants from each of the four years at university (*n* = 20), FYPs (*n* = 5) and NQPs (*n* = 5). Each pharmacy student researcher was allocated a specific cohort to interview. Despite criticisms of defining a sample size *a priori* [32], a maximum sample of 30 was decided to manage the students’ workloads and was considered appropriate as it is broadly within accepted practice for sample sizes for this research [32].

Participants were recruited using adverts (designed by the pharmacy student researchers) via schools of pharmacy online student portals and social media platforms (X and Instagram). Snowball sampling was deployed as adverts could be liked, shared and reposted, and other students were asked to share the information with their peers. A £15 voucher was offered to participants as a gesture of appreciation. Owing to the nature of social media recruitment and the financial incentive, participant eligibility was confirmed based on their use of a university email address or inclusion on the GPhC list of registered pharmacists.

### Data collection and analysis

To prepare for data collection and analysis, the pharmacy student researchers undertook methods training by the lead researchers (E.D. and N.W.), with one session on interviewing techniques and another on data analysis. Lead researcher E.D. conducted a pilot interview, and the recording of this interview was then used to train the student researchers. The lead researchers then coached the pharmacy student researchers on interview techniques as well as inductive thematic data analysis, using NVivo (Lumivero, Denver, CO, USA).

The six pharmacy student researchers conducted the one‐to‐one semi‐structured interviews online via Microsoft Teams (Microsoft, Redmond, WA, USA) between November 2023 and December 2023. Interviews lasted between 17 and 60 minutes and were audio‐ and video‐recorded and transcribed via the platform’s inbuilt technology, before being pseudo‐anonymised and edited for accuracy using an intelligent verbatim approach [33]. Each pharmacy student researcher conducted inductive thematic analysis of the transcripts from the interviews, as described by Braun and Clarke (2006) [33], assisted by NVivo. The thematic analysis process involved the following steps: researcher familiarisation with the data (i.e. students reading the transcripts of their own interviews); initial inductive coding; grouping codes to identify key themes and sub‐themes (which were discussed at group meetings), with relevant portions of the data charted against each other; reviewing the themes (iteratively at group meetings); defining and naming themes/sub‐themes; and individual reporting through student dissertations.

Any written notes made by student researchers retained confidentiality and were not incorporated into the analysis. To maintain consistency in the analytical approach, all students attended weekly 2‐hour meetings over several weeks with the lead researchers, where the interim analysis was discussed, including a discussion of coding and associated quotes from the transcripts. The lead researchers E.D. and N.W. (experienced in qualitative research) alongside A.M. and A.R. reviewed the themes and collectively developed a final thematic map. Transcripts were not returned to participants and they were not asked to feedback on the findings, as this study was conducted during university exam periods, which impacted most participants, and the researchers wanted to minimise any disruption to their studies.

### Ethics

Ethical approval was granted by the Strathclyde Institute of Pharmacy & Biomedical Sciences (SIPBS) Departmental Ethics committee.

### Researcher reflexivity

Interviews were conducted by the pharmacy student researchers (of both male and female genders) who had no previous interview experience, and hence the pilot interview was used to train the student researchers. The lead researchers coached them on interview techniques (i.e. open‐ended questions and prompting), the role of a researcher in conducting the interview (and how this differs from consulting with patients as student pharmacists), and the importance of impartiality and minimising bias. On two occasions the student researchers had pre‐existing relationships with participants, which was not unexpected given the researchers were interviewing participants from their home institution. On these two occasions, another researcher without a prior relationship conducted the interviews instead to minimise bias. At the time of the study, the lead researchers E.D. (MPhil) and N.W. (PhD) each worked at the University of Strathclyde as a researcher and lecturer, respectively, A.R. (PhD) worked as a pharmacist in NHS Tayside, and A.M. (BSc) and T.B. (MPH) worked at Healthcare Improvement Scotland within the Justice area. Therefore, as the authors predominately represent the pharmacy profession, they may have had biases/assumptions about the research topic in terms of pharmacy professionalism and the care of this patient population.

## RESULTS

Forty‐eight people expressed interest in participating. Based on participant availability to attend an interview, and the purposive sampling methodology employed (i.e. an intended sample of five participants from each of the four years of study at university plus FYPs and NQPs; representation from both schools of pharmacy; and variation in demographic characteristics), 29 participants were ultimately invited to interview. One participant did not attend for reasons unknown, resulting in 28 participants. The majority were female (*n* = 22, 78.6%) and attended the University of Strathclyde (*n* = 21, 75.0%). All participants had experience within pharmacies that provided OAT and services for nicotine dependency. The full demographics are presented in Table 1.

**TABLE 1:** Participant demographics.

Demographics	*n*	%
Gender		
Female	22	78.6%
Male	5	17.9%
Non‐binary	1	3.6%
Ethnicity (self reported)		
White/British/Scottish/Irish	24	85.7%
Mixed White/Asian	2	7.1%
Chinese	1	3.6%
Black	1	3.6%
Participant group		
Year 1 student	5	17.9%
Year 2 student	5	17.9%
Year 3 student	5	17.9%
Year 4 student (final year)	5	17.9%
Foundation Year Pharmacist (FYP)	2	7.1%
Newly Qualified Pharmacist (NQP)	6	21.4%
University of Study		
University of Strathclyde	21	75.0%
Robert Gordon University	7	25.0%
Dependency harm reduction services in pharmacies in which participants had experience		
Opioid agonist therapy (OAT) (i.e. methadone/buprenorphine supervision and supply)	28	100.0%
Services for nicotine dependency (e.g. supply of nicotine patches)	28	100.0%
Needle and syringe services	15	53.6%
Naloxone supply	13	46.4%
Services for alcohol dependency (e.g. alcohol brief intervention and supervisions)	11	39.3%
Buvidal® administration	2	7.1%
Blood‐borne virus testing	1	3.6%
Health boards worked in^a^		
NHS Greater Glasgow & Clyde	15	53.6%
NHS Lanarkshire	12	42.9%
NHS Lothian	10	35.7%
NHS Ayrshire & Arran	8	28.6%
NHS Grampian	8	28.6%
NHS Dumfries and Galloway	2	7.1%
NHS Fife	2	7.1%
NHS Forth Valley	2	7.1%
NHS Highland	2	7.1%
NHS Borders	1	3.6%
NHS Orkney	1	3.6%
NHS Tayside	1	3.6%

^a^ Most participants had experience working in community pharmacies in more than one health board.

### Theme 1: public stigma and insufficient care

#### Stigma in the community pharmacy

All participants across all groups witnessed some extent of stigma towards people with substance dependency by community pharmacy staff. Often, participants had observed a negative culture lacking compassion toward this patient population, with pharmacy staff being observed to question the legitimacy and worthiness of healthcare provided to people with substance dependency, compared with other patient groups, with some reference to their healthcare needs being self‐inflicted. Hesitancy to support this patient group and to overlook their potential for recovery was also observed, with one participant stating that they had encountered staff who believed people with substance dependency were ‘too far gone’ (Year 3, P5):

‘They’re just seen as lower class and like they got themselves into that situation as if it’s their fault. And actually, no one looks at the bigger picture’ (NQP, P4)

Participants commonly reported prejudice around people with substance dependency as a group, owing to stereotyping, which they felt created an environment where staff and other patients separated themselves from people with substance dependency as they are ‘seemingly dangerous’ (Year 4, P2) or more likely to engage in criminal activity in the pharmacy. Stigma from other patients (i.e. the public) was also reported as prevalent, which included negative language used about people with substance dependency and stigmatising behaviours, which resulted in natural segregation:

‘The general public who are in the shop will tend to avoid the substance dependency patients’ (Year 1, P2)

Most participants reported their discomfort with this and with the derogatory language used, which was perceived as depersonalising:

‘I actually loathe terms like “junkies”. I know, you say it colloquially. A lot of people do it because it’s just an easy way of referring to them. But at the end of the day, they are people, and they are patients’ (NQP, P3)

#### Negative care observed

Most participants shared examples of how they observed people with substance dependency being treated differently to other patient groups by community pharmacy staff. For example, people with substance dependency may be asked to wait in a separate area in the community pharmacy, were rushed out or, conversely, were considered less of a priority and made to wait for longer, which in one instance was perceived as deliberate:

‘I’ve worked with quite a strict pharmacist too and they made them wait, so it was that they had to get a day [of treatment] removed or something and they were made to wait for half an hour … to make a point’ (Year 1, P3)

The general attitude towards people with substance dependency was considered negative, with disrespectful comments made by the pharmacy staff when people with substance dependency were not in the pharmacy, and participants witnessed community pharmacy staff lacking a friendly demeanour:

‘[The staff] are a bit more bitchy about the patients and I think that comes across when they serve them as well, just even their mannerisms, being a bit harsh … not politely handing it over like you would another prescription’ (NQP, P1)

There were also instances where it was observed that fundamental principles of professional practice were not adhered to, such as a lack of counselling about treatments and goal setting for people with substance dependency, with treatment not well explained. Lack of confidentiality was another example provided by a smaller number of participants, whereby supervised consumption was not done in private:

‘When I became a locum, I realised that a lot of the shops do not have privacy for methadone supervision. It’s totally inappropriate. Expecting someone to even drink methadone over the counter is beyond belief. I think that’s ridiculous’ (NQP P1)

### Theme 2: structural stigma of barriers to accessing care

Almost all FYP/NQP participants reported not feeling equipped or connected enough with other services to be able to offer people with substance dependency the support they need, and it was remarked that there was limited information on what services were available for people with substance dependency and the routes for referral. Some participants commented that other healthcare professionals like general practitioners (GPs) do not always know how best to support people with substance dependency, and at times patients were sent back and forth to the pharmacy:

‘[people with substance dependency] often get referred back to the pharmacy because [GPs] want to kick it back to you to deal with … It’s hard to get others like the [multidisciplinary team] basically to take the patients seriously’ (NQP, P1)

A smaller number of participants reported barriers to accessing care and support, particularly out of hours, which left both pharmacy staff and people with substance dependency frustrated. It was reported that Community Addiction Teams were not available out of hours, with an example provided of a patient not able to access treatment over the weekend reporting they would ‘need to go and use now’ (FYP, P3) as a result. OAT was reportedly not prescribed by the health service out of hours in Scotland, resulting in pharmacy staff feeling unsupported to help people with substance dependency:

‘You used to be able to contact NHS 24 for support but now they just will not prescribe anything. They won’t prescribe methadone or buprenorphine out of hours, so I do feel like there is no support for a pharmacist or pharmacy staff in that situation at the weekend’ (FYP, P3) (NHS 24 is Scotland’s national telehealth and telecare organisation that supports patient care out of hours.)

### Theme 3: impact of observations on professional identity formation

#### Positive care showcasing the role of community pharmacy

The impact of positive experiences between pharmacists and people with substance dependency was discussed, which gave many participants insight into the role community pharmacy can play. Fundamentals to cementing a positive relationship with people with substance dependency was creating a secure and transparent environment, symbolising safeguarding. A small number of participants reported that people with substance dependency greatly appreciated when they were treated with respect, attributing this to people with substance dependency feeling comfortable to talk about ‘how they feel about things and their concerns’ (NQP, P1). It was reported that trusting their pharmacist was crucial for people with substance dependency, particularly when the pharmacist consistently works in the same pharmacy, which results in building rapport with both the patient and their family:

‘I do think a lot of people just in general, not only [people with substance dependency], a lot of people do trust the pharmacist. I would say them more though because of the regular contact, you know you’ll build rapport with patients and their close family as well’ (Year 2, P3).

Some participants observed the valuable impact a pharmacist can have on a patient’s treatment journey. Owing to the nature of treating people with substance dependency, it was reported that many of these patients are interacting with the pharmacy daily and when treated with respect and care this can be greatly beneficial. On the contrary, if the care they receive is not of a high standard participants considered this to also have a detrimental effect:

‘I didn’t quite realise how big a part, or the impact that you can personally have on one person’s life because you see them every day is massive. If you do it correctly, equally you can have an amazing impact, but you can also have a dreadful impact if you do not do it correctly’ (Year 4, P5)

#### Influence that observing pharmacists has on student beliefs and behaviour

It was common for participants to report that observing high levels of professionalism influenced their own attitudes and behaviours. Participants reported being reliant on observing the mannerisms of the pharmacist to shape their own behaviours, and reported that the professionalism of pharmacists, both positive and negative, helped create their own professional identity. Some participants had worked with a variety of pharmacists, stating that no pharmacist works the same, and most believed this helped them shape a positive professional identity:

‘I’ve got so many pharmacist role models that I’ve met throughout being in pharmacy that you try and pull everything from them to try and make you the best you can be’ (NQP, P4)

Pharmacists offering holistic care and social support for people with substance dependency were considered admirable, including pharmacists being involved in wider care such as social care aspects and contacting GPs on behalf of patients. Participants in earlier years of their pharmacy education had initially believed that supplying OAT to people with substance dependency was a quick encounter, with the focus on handing out the medication. When observing pharmacists, some participants realised the importance of patient education and signposting to services, and the role they could have in ‘looking out’ for patients:

‘But I want to be the type of pharmacist that can kind of gauge how they’re feeling overall or “aw like I noticed you’re feeling a bit different, is there anything I could help you with?” things like that. So, I think it’s made me realise how important it is for me to be empathetic and how I want to be the type of pharmacist that listens to my patients’ (Year 4, P2)

Participants often referred to poorer quality care that they had observed, with judgement on how this was not favourable in their view, using words such as ‘unacceptable’ (NQP, P2) or referring to a pharmacy staff member’s attitudes as ‘bitchy’ (FYP, P1). In some instances, this evidently influenced their own chosen demeanour:

‘Maybe there’s been a few locums actually, who have been quite short with patients, and I think that’s not good. I think that you should try to be friendly.’ (Year 2, P2) (In Scotland, ‘short’ is a colloquial term referring to someone who is abrupt in demeanour.)

### Theme 4: preparation for professional practice

#### Insufficient education

A subset of participants, mostly those within early years of their pharmacy education, showed uncertainty about their knowledge of people with substance dependency and their treatment, and there was a degree of apprehension when working with people with substance dependency. Participants felt unprepared to support people with substance dependency, with reference to people with substance dependency not being considered a ‘priority at all’ (NQP, P2), and with participants’ education focusing mostly on the mechanism of action of treatment:

‘I don't feel like the university, or the coursework on my foundation year training year, I don’t feel like that had any benefit to treating these types of people because that was all very like, this is what methadone is, this is how it works. This is buprenorphine, how it works’ (FYP, P1)

There was reference to little course content on how to support people with substance dependency, with the sensitivities not emphasised sufficiently in formal education:

‘I do think we did learn a lot about, you know, about the substance misuse side. But learning about the other care issues that may come along with that I think have been lacking’ (Year 4, P5)

Recommendations were posed by some participants as to how to best prepare them to support people with substance dependency. This mostly related to: content on the treatment of people with substance dependency earlier on in the pharmacy degree; providing education on how to signpost to information and recovery routes; more experiential learning; having people with lived experience of substance dependency participating in teaching; and focusing education on prevention and harm reduction. Overall, this generally related to the need for a more holistic approach to university‐taught material in relation to this patient population:

‘I think more could be taught about, well, why do people in deprived areas, why are they more likely to become unwell? Or why does this exist? Just more about health inequalities in general and more teaching as to why it exists’ (Year 2, P3)

#### The importance of experience

Some participants also felt there was differentiation between what they learned at university compared with what they experienced in practice, with one participant stating that ‘uni makes pharmacy more professional than I’ve experienced in community pharmacy’ (Year 1, P3). There was value attributed to working within community pharmacy practice, with reference to experiential learning where students would apply their knowledge, which positively impacted their development. There appeared to be consensus that it was this experience, and not university teaching, which best prepared participants to interact with people with substance dependency, namely in building their confidence, addressing shyness and establishing their values:

‘I think university gave me a good starting point, but actually seeing it in practice is what made me change my values, change the way I treat people as well’ (Year 4, P2)

‘My work has really helped me because probably before I started or just as I started, I was quite shy … I definitely didn’t just talk to anybody at the start so confidently. Now, when they [people with substance dependency] come in every week, no matter who the patient is, I’ll be like “oh hi, how are you?”. So, I feel like that’s kind of just the soft skills that you don’t really see in university as much’ (Year 2, P4)

## DISCUSSION

This study provides a fresh narrative of how the care of people with substance dependency in Scottish community pharmacies is perceived by students and NQPs. Community pharmacies have been positioned as the first port of call for healthcare in the UK through the implementation of services such as Pharmacy First [34, 35]. Community pharmacy staff engage with people with substance dependency far more frequently than other professional services through various harm reduction initiatives across the UK and internationally [5]. This study found evidence of the ostracising of people with substance dependency, with some pharmacy team members treating this patient population differently to others. There was also evidence that healthcare policies, procedures and operational routines created systematic bias and stigmatisation, acting as a barrier to accessing healthcare. Conversely, there was evidence of exemplary practice, with some pharmacy staff offering holistic and compassionate longitudinal care. This observed variation in practice impacted participants’ professional identity formation and their vision of the pharmacists they aspired to become.

### Discussion of observed practice

The negative and stigmatising treatment of people with substance dependency has been identified previously within Scotland and from an international review of qualitative literature [36, 37, 38], and may be explained by the beliefs, attitudes and ethos existing within pharmacy teams. From the perspective of patients, Radley *et al*. similarly identified people with substance dependency being treated differently and adversely within Scottish community pharmacies, which perpetuated notions of social isolation [36], and echoed findings of a review on global experiences [37]. A 2022 overview of systematic reviews also identified social stigma as a key barrier to substance misuse recovery [39]. This study additionally found evidence that pharmacies may not have adequate resources to provide a positive and confidential care experience, resulting in policies and practices that likely influence the experience of people with substance dependency [40]. The lack of integration with the wider multi‐disciplinary team and limited support available to pharmacy teams will likely contribute to the deviation of practice from norms adhered to by other health professionals, as isolated practice may promote the adoption of different behaviours, and limited integration of pharmacy teams within multi‐disciplinary care planning may hamper the promotion of recovery activities and support [38].

The stigmatisation of people with substance dependency may affect their health‐seeking behaviour and trust in healthcare professionals, contributing to medication non‐adherence, the exacerbation of health conditions, the economic burden and a compounding of health inequalities [19, 41]. The findings of this study exemplify some challenges facing people who may experience social exclusion from mainstream society as a result of substance dependence, with indications that the pharmacy care provided is lacking the necessary holistic approach and consideration of broader factors [42]. This could jeopardise recovery and prolong the adverse consequences of poverty and exclusion that underlie the health inequalities that are observed [43]. Recovery from addiction is a socially mediated process, and recognising and addressing stigma is vital for fostering social justice, equality and effective service delivery within a community [41, 44, 45], which requires non‐judgemental healthcare that recognises the experience of social exclusion [42]. Community pharmacies should provide interventions and treatments that support a recovery journey, acknowledging the importance of social connections, a recovery identity and citizenship (i.e. reclaiming a place in society) [46]. Part of the recovery journey is the recognition provided by being treated with dignity and respect, which has been identified as the most sought‐after attribute from community pharmacy services [18, 42]. There is a need to implement educational interventions within pharmacy settings that address stigmatising attitudes [47]. This should ensure that the provision of care adheres to international standards for the treatment of substance dependency, by reducing the burden of stigma, and the fundamental right to access to high quality healthcare [48, 49].

### Students’ professional identity formation

Student participants valued exposure to a range of behaviours, and provided rich narratives and reflection of their own attitudes [50]. It is evident that all types of observations, good and bad, can shape professional identity positively – allowing students to explore and enact their identity as a pharmacist [23, 51]. The importance of experiential learning on the development of a pharmacy student’s identity has been well established [23]; however, not all experiential learning programmes have a focus on professionalism [52]. Additionally, our study suggests the importance of gaining experience in a wide range of settings, as there is a risk that experience gained in pharmacies lacking in professionalism could prevent a true understanding of the broad scope of practice and perpetuate existing cultures of stigma and discrimination. Educators in pharmacy practice may wish to evaluate the range of experiences that students are exposed to through the lens of student pharmacists to identify the professionalism observed and consider the recommendations posed, particularly relating to vulnerable populations in society.

The reported insufficiency of undergraduate education in preparing pharmacists was a prominent finding, with improved healthcare treatment provider training and structures also identified by Farhoudian *et al*. (2022) as a key facilitator to substance use recovery [39]. Pharmacy undergraduate curricula are pressured to accommodate increasing volumes of technical and practice‐based content. In terms of didactic knowledge, pharmacy education across the globe currently has no agreed evidence‐based criteria determining which foundational concepts are most important and no assessment tools to monitor progress in learning [53], and in general there is limited international research on this topic [53]. Given these constraints, pharmacy curricula must ensure fundamental concepts and ethical perspectives necessary for professional practice are firmly embedded [54], with a curriculum that prioritises professional identity formation [23]. Furthermore, there should be consideration of the cognitive frameworks with which students are provided, which could influence their cognitive process, whereby taught and observed practice informs their professional beliefs and behaviour. Although this study did not uncover the in‐depth cognitive process from which observations influence beliefs and then behaviours, future research may wish to do so guided by theories of identity formation.

### Novelty and implications of the research

These results show our first attempt in understanding the benefits and constraints of experiential learning in pharmacy education from a student perspective, using peer student researchers. This approach draws upon the ‘mystery shopper’ approach widely employed as a form of participant observation to gauge the acceptability of service provision [55]. It has allowed the research team to think creatively about the development of methodologies for practice evaluation and assessment of the quality of experiential placements, as direct monitoring of this is difficult to achieve. Providing students with a voice, alongside a context and reporting structure, may have learning benefits in terms of increasing their ability to discriminate between desirable and poorer practice. The ability of students to do so will contribute to professionalism, which is likely to improve patient outcomes as well as the quality of new pharmacy professionals. Additionally, the involvement of pharmacy undergraduates as researchers in data collection and analysis using a structured research protocol is likely to provide important orientation and exposure to research participation and, it is hoped, may further their participation in research when they qualify. This is of particular importance as research is increasingly recognised as an integral part of the role of a pharmacist [26]. Despite a global drive to transform pharmacy practice by increasing research capacity within the profession [56, 57, 58], competency in research is deficient for many practitioners [59], with a need to improve research readiness and capacity to engage [60, 61, 62].

### Strengths and limitations

This study enabled undergraduate students to conduct a formal research study and to analyse the qualitative interviews of, in part, their peers. A particular strength is therefore the interpretation of results from the perspective of the group going through the experiential learning placement. Additionally, students and early career pharmacist participants may have felt more comfortable to share their honest opinion when speaking to the pharmacy student researchers, rather than to an academic or qualified pharmacist. Fewer participants were recruited from Robert Gordon University than hoped, and the difference in exam timetables between the two schools of pharmacy in Scotland may have influenced recruitment. Although there was no formal assessment of data saturation, through iterative discussions between the lead and student researchers that occurred weekly it was apparent that similar concepts were arising across interviews [63]. Furthermore, the data from the 28 participants were considered sufficient to provide in‐depth understanding, and the sample size was within recommendations [32]. However, future research could be expanded to more diverse pharmacy settings, to enhance the transferability of the findings, and would benefit from capturing demographic factors, which may influence participants’ experiences and opinions. Additionally, future studies may wish to treat each year of education as a single cohort, which would enable cross comparison to understand the nuances in professional identity formation between those at different stages in their pharmacy education. A potential limitation was the group approach to data analysis. Although several hours of group discussion and iterative analysis occurred, a single‐analysist approach, led by one researcher and validated by another, may have improved consistency. Additionally, as the student researchers and the authors of this article predominately reflect the pharmacy profession, this poses a risk of subconscious reporting bias. However, having multiple people involved in data analysis limits the likelihood of this. Finally, the use of a theoretical framework via deductive analysis could have assisted with the consistency of coding; however, this may have resulted in divergent or unexpected themes being missed.

## CONCLUSION

This study utilised peer student researchers to interview student and early career pharmacists about their experiential placements, exploring the development of their professional identity in the context of caring for people with substance dependency. This study revealed that stigma and lack of professionalism in pharmacy practice can have a negative impact on trainee pharmacists, which may in turn affect how care is delivered to this vulnerable population. Pharmacists acting as role models have the power to shift this and promote the destigmatisation and acceptance of people with substance dependency. This research reinforces the importance of varied experiential learning, the inclusion of professional identity formation in undergraduate curricula and the need for insufficient care in community pharmacy to be addressed, with a focus on how social inclusion can be fostered. The insights provided in this study, together with the enthusiastic participation of the student researchers, provides support to repeat the exercise in further areas of interest and more diverse pharmacy settings. Exploring stigmatising practises in pharmacy is crucial considering how it may impact recovery, and future research should explore interventions that can address stigmatising attitudes in practice [47] to ensure the fundamental right to high‐quality healthcare is achieved [48, 49].

## AUTHOR CONTRIBUTIONS

**Natalie Weir:** Conceptualization (equal); formal analysis (equal); funding acquisition (equal); methodology (equal); project administration (equal); resources (equal); supervision (equal); validation (equal); visualization (equal); writing—original draft (equal); writing—review and editing (equal). **Emma Dunlop:** Conceptualization (equal); formal analysis (equal); funding acquisition (equal); methodology (equal); project administration (equal); resources (equal); supervision (equal); validation (equal); visualization (equal); writing—original draft (equal); writing—review and editing (equal). **Adrian MacKenzie:** Conceptualization (equal); formal analysis (equal); funding acquisition (equal); methodology (equal); supervision (equal); validation (equal); visualization (equal); writing—review and editing (equal). **Thomas Byrne:** Conceptualization (equal); formal analysis (equal); validation (equal); visualization (equal); writing—review and editing (equal). **Katie Johnston:** Data curation (equal); formal analysis (equal); investigation (equal); validation (equal); visualization (equal); writing—review and editing (equal). **Alice O'Hagan:** Data curation (equal); formal analysis (equal); investigation (equal); validation (equal); visualization (equal); writing—review and editing (equal). **Zohaib Rehman:** Data curation (equal); formal analysis (equal); investigation (equal); validation (equal); visualization (equal); writing—review and editing (equal). **Holly Richardson:** Data curation (equal); formal analysis (equal); investigation (equal); validation (equal); visualization (equal); writing—review and editing (equal). **Aalia Shah:** Data curation (equal); formal analysis (equal); validation (equal); visualization (equal); writing—review and editing (equal). **Gemma Wilson:** Data curation (equal); formal analysis (equal); investigation (equal); validation (equal); visualization (equal); writing—review and editing (equal). **Andrew Radley:** Conceptualization (equal); formal analysis (equal); funding acquisition (equal); methodology (equal); supervision (equal); validation (equal); visualization (equal); writing—original draft (equal); writing—review and editing (equal).

## DECLARATION OF INTERESTS

None to declare.

## Supporting information


**Appendix S1.** Consolidated criteria for reporting qualitative studies (COREQ): 32‐item checklist.

**Appendix S2.** Interview schedule.

## Data Availability

Research data are not shared.
