# Supplementary material for: Professionalism, professional identity and community pharmacy culture: The context of substance dependency through the lens of student and early career pharmacists
Source: Addiction. 2025 Sep 25;121(1):138–49. doi: 10.1111/add.70180 (PMC12710688; doi:10.1111/add.70180)
Supplement: Supplementary file 2 — Appendix S2. Interview schedule. [file ADD-121-138-s002.docx]

**Appendix 2: Interview schedule**

Perceptions of professionalism (core values, core norms)

1. Tell us about your experience of working with people with substance dependency within your experiential learning / your practice?
   1. How did you interact with them?
   2. How did other staff interact with them?
2. What did/do you think about the culture in the pharmacies you have been/are in when working with people with substance dependency?
   1. **Trustworthiness** - do you think the expectations of pharmacy were/are made clear to people with substance dependency? Why, why not?
   2. **Safety**- Is/was effort made to make the pharmacy feel like a safe space (physically and emotionally? Prompts: stigma, confidentially etc
   3. **Collaboration** – to what extent did/do the pharmacy link with other organisations or sources of support? Prompts: referrals, social care etc
   4. **Choice** and **empowerment** – To what extent were/are peoples’ voices heard? To what extent was/is that voice an active part in the decisions / care received?
   5. Thinking about your experience in the different pharmacies you have been in. To what extent did/do pharmacies differ / compare / contrast in relation to this?

Professional identity (internalisation of professionalism)

1. To what extent has your experiential learning / practice influenced your perceptions of pharmacists in seeing patients with substance dependency?
   1. The **role** of the pharmacy profession
   2. The **responsibilities** of the pharmacy profession
   3. The **values** of the pharmacy profession
   4. The **ethical standards** of the pharmacy profession
2. To what extent has your EL / practice influenced your thoughts on what kind of pharmacist you aspire to be?
   1. Your **role** as a pharmacist / aspiring pharmacist
   2. Your **responsibilities** as a pharmacist / aspiring pharmacist
   3. Your **values** as a pharmacist / aspiring pharmacist
   4. Your **ethical** standards as a pharmacist / aspiring pharmacist

Teaching and Training

1. To what extent did your training prepare you for interacting with people with substance dependency in your EL / practice?
   1. Why / why not / what was most / least relevant?
2. What, if anything, do you think you would need to learn to support people with substance dependency moving forward?
   1. What to take forward?
   2. Where are the gaps?
3. Think about your perceptions of what it means to be a pharmacist - to what extent has this evolved since you began university until now?
   1. Think about substance dependency in your response
   2. More generally (if relevant)
4. Anything else / other questions?
